# Reciprocally-Benefited Secure Transmission for Spectrum Sensing-Based Cognitive Radio Sensor Networks

**DOI:** 10.3390/s16121998

**Published:** 2016-11-25

**Authors:** Dawei Wang, Pinyi Ren, Qinghe Du, Li Sun, Yichen Wang

**Affiliations:** 1 Department of Information and Communication Engineering, School of Electronic and Information Engineering, Xi’an Jiaotong University, Xi’an 710049, China; wangdw@stu.xjtu.edu.cn (D.W.); duqinghe@mail.xjtu.edu.cn (Q.D.); lisun@mail.xjtu.edu.cn (L.S.); wangyichen0819@mail.xjtu.edu.cn (Y.W.); 2 Shaanxi Smart Networks and Ubiquitous Access Research Center, Xi’an 710049, China; 3 Key Laboratory of Wireless Sensor Network & Communication, Shanghai Institute of Microsystem and Information Technology, Chinese Academy of Sciences, 865 Changning Road, Shanghai 200050, China

**Keywords:** cognitive radio sensor network, secrecy outage probability, spectrum sensing error, power allocation

## Abstract

The rapid proliferation of independently-designed and -deployed wireless sensor networks extremely crowds the wireless spectrum and promotes the emergence of cognitive radio sensor networks (CRSN). In CRSN, the sensor node (SN) can make full use of the unutilized licensed spectrum, and the spectrum efficiency is greatly improved. However, inevitable spectrum sensing errors will adversely interfere with the primary transmission, which may result in primary transmission outage. To compensate the adverse effect of spectrum sensing errors, we propose a reciprocally-benefited secure transmission strategy, in which SN’s interference to the eavesdropper is employed to protect the primary confidential messages while the CRSN is also rewarded with a loose spectrum sensing error probability constraint. Specifically, according to the spectrum sensing results and primary users’ activities, there are four system states in this strategy. For each state, we analyze the primary secrecy rate and the SN’s transmission rate by taking into account the spectrum sensing errors. Then, the SN’s transmit power is optimally allocated for each state so that the average transmission rate of CRSN is maximized under the constraint of the primary maximum permitted secrecy outage probability. In addition, the performance tradeoff between the transmission rate of CRSN and the primary secrecy outage probability is investigated. Moreover, we analyze the primary secrecy rate for the asymptotic scenarios and derive the closed-form expression of the SN’s transmission outage probability. Simulation results show that: (1) the performance of the SN’s average throughput in the proposed strategy outperforms the conventional overlay strategy; (2) both the primary network and CRSN benefit from the proposed strategy.

## 1. Introduction

Wireless sensor networks have been identified as a promising technology for the ongoing developing intelligent world and are widely deployed for different application fields, such as military, environmental monitoring [1], healthcare [2], smart home [3] and other commercial areas [4]. By taking advantage of the characteristics of the self-organization and flexible expansion [5,6], wireless sensor networks are widely utilized for wireless monitoring and controlling, especially for the smart home system. However, thousands of sophisticated, overlapping and coexisting wireless sensor networks burden the limited licensed spectrum, where sensor nodes (SN) suffer from strong interference. In addition, the license-exempt industrial, scientific and medical (ISM) bands are crowded with other communication systems, such as WiFi, wireless local area networks (WLANs), Bluetooth, etc. [7]. Therefore, to improve the performance of the wireless sensor networks, making full use of the limited wireless spectrum is a critical issue.

Cognitive radio technology, initially proposed by Mitola [8], greatly relieves the spectrum shortage situation and improves the spectrum efficiency. In cognitive radio networks, the secondary system can share the licensed spectrum through underlay or overlay modes. For the underlay spectrum sharing mode, the secondary network accesses the licensed spectrum concurrently with the transmission of the primary network under the interference temperature constraint. For the overlay spectrum sharing mode, the secondary system detects the unutilized licensed spectrum (known as white space) to access and avoids interference to the primary network. As a promising spectrum sharing approach, the overlay mode, which not only improves the throughput of the secondary network, but also guarantees the quality of service (QoS) requirement of the primary network, will be adopted in this paper. The energy detection technique is mostly utilized in the overlay mode to be aware of the available surrounding spectrum resource [9]. However, since the inevitable spectrum sensing errors will result in the performance degradation of both the primary and secondary networks, the network parameters should be carefully designed to improve the spectrum sensing accuracy [10].

Considering the strong interference from other communication networks in ISM bands and the QoS requirements for applications, wireless sensor networks can integrate the cognitive radio technology and formulate the cognitive radio sensor network (CRSN) to efficiently utilize the spectrum [11,12]. Therefore, the information in the CRSN can be efficiently and reliably transmitted in the unutilized licensed spectrum. Recently, CRSN has attracted much research attention [13,14,15,16,17,18,19]. An optimal schedule scheme for a sensor-aided energy-efficient cooperative network was designed in [13]. An energy-efficient channel management scheme was proposed in [14] to save the energy for spectrum sensing and prolong the network lifetime. In [15], the authors designed a spectrum-aware medium access control (MAC) protocol for CRSN. In [16], the authors analyzed the delay performance and designed two channel switch methods for CRSN. The work [17] investigated the modeling methods for information transmission, and [18] analyzed the spectrum efficiency through a graph-theoretic max-flow framework for CRSN. Spectrum and power allocation and routing were jointly considered to maximize the information rate, as well as the lifetime of CRSN [19]. However, in the above works, the effects of the spectrum sensing errors are not considered. Since the inevitable spectrum sensing errors will bring strong interference to the transmission of the primary users (PU), the spectrum sensing accuracy should be improved. Although much more time allocated for the spectrum sensing can improve the spectrum sensing accuracy, there will be less time for SN’s transmission, which will decrease the throughput of the sensor network. Therefore, there is a tradeoff between the spectrum sensing accuracy and the QoS provisioning of the CRSN.

Due to the broadcast nature of wireless media, the primary network faces the eavesdropping threat, as all other users in the primary transmission range are potential eavesdroppers. Traditionally, the upper layer encryption and decryption algorithms, assuming limited computational ability at the eavesdropper, are adopted to guarantee the information security. Besides, by employing the characteristics of wireless channels, physical layer security methods, such as cooperative relaying and/or jamming, can also provide perfect information security for the primary confidential messages [20,21]. Through being assisted by one or multiple jammers, cooperative jamming is a simple way to protect the confidential messages against eavesdropping [22,23,24]. The work in [25] proposed a destination-aided jamming scheme to protect the confidential messages against the untrusted relay. The work in [26] optimally designed the precoding matrix and allocated the transmit power of the source and jammer to maximize the lower-bound secrecy rate in a multi-input multi-output cognitive radio network. In [27], a cooperative node was chosen to act as a cooperative jammer to transmit jamming signals or act as a noise forwarder to transmit dummy codewords. In [28], the secondary destination and transmitter transmitted jamming signals during the primary broadcasting and forwarding phases, respectively, to guarantee the security of the primary information. In [29], the primary system would cooperate with either two secondary users or a cluster of secondary users to improve its secrecy rate. The authors in [30] proposed a cooperative jamming scheme in which the secondary system transmitted the jamming signal to acquire some spectrum opportunities as a reward. In CRSN, we can also utilize the physical layer approach to protect the confidential messages. In addition, we notice that in CRSN, the transmission of the SN also interferes with the eavesdropper, which can be employed to enhance the primary secrecy rate. Therefore, in this paper, we will utilize the interference from the SN to protect the primary confidential messages, and as a reward, the limited spectrum sensing error probability of the CRSN is permitted to satisfy the SN’s QoS provisioning. To the best of the authors’ knowledge, this is the first work that has investigated the secure transmission of the primary system assisted by CRSN by taking into account the spectrum sensing errors.

In this paper, we propose a reciprocally-benefited secure transmission strategy to protect the primary confidential messages and support the QoS requirement of CRSN. In the proposed strategy, the SN opportunistically shares the unutilized licensed spectrum through spectrum sensing. Due to the inevitable spectrum sensing errors, the primary transmission are interfered by the transmission of the SN. In addition, the primary network faces the security threat from an eavesdropper. Since the interference from the SN also interferes with the eavesdropper, which can be employed to increase the primary secrecy rate, the primary network can permit limited spectrum sensing error probability in exchange for the SN’s cooperation. Both CRSN and the primary network can benefit from the cooperation. From the primary service perspective, this transmission strategy transforms the possible disturbed SN’s service activities into a reciprocally-benefited mode, and the primary confidential messages are protected. From the SN’s service perspective, limited spectrum sensing error probability is permitted, and the SN’s QoS is satisfied. Specifically, according to the PUs’ activities and spectrum sensing results, there are four system states: (I) the spectrum is idle and detected as idle; (II) the spectrum is occupied and detected as idle; (III) the spectrum is idle and detected as busy; and (IV) the spectrum is occupied and detected as busy. For each state, we analyze the SN’s transmission rate and the PUs’ secrecy rate. Then, we optimally allocate the SN’s transmit power for each state to maximize the average transmission rate of CRSN under the constraints of the SN’s average transmission power and the PUs’ secrecy outage probability requirements. We give a further discussion for the power allocation scheme and investigate the performance tradeoff between the transmission rate of CRSN and the secrecy outage probability of the primary network. In addition, we analyze the PUs’ secrecy rate for the asymptotic scenarios and derive closed-form expression of the SN’s transmission outage probability. Extensive simulations are implemented to evaluate the proposed strategy. We investigate the effects of the spectrum sensing time, target detection probability and the PUs’ secrecy outage probability on the SN’s average throughput. Simulation results show that: (1) the performance of the SN’s average throughput in the proposed strategy outperforms the conventional overlay strategy; (2) the primary confidential messages will be protected, and the SN can access the licensed spectrum with permitting limited spectrum sensing error probability.

The rest of this paper is organized as follows. Section 2 describes the system model, as well as the four system states. Section 3 interprets the proposed strategy. The optimal power allocation problem is formulated and solved in this section. In Section 4, we give a further discussion for the power allocation scheme and investigate the performance tradeoff between the transmission rate of CRSN and the secrecy outage probability of the primary network. We conduct extensive simulations in Section 5, and Section 6 concludes the paper.

## 2. System Model

**Figure 1:**
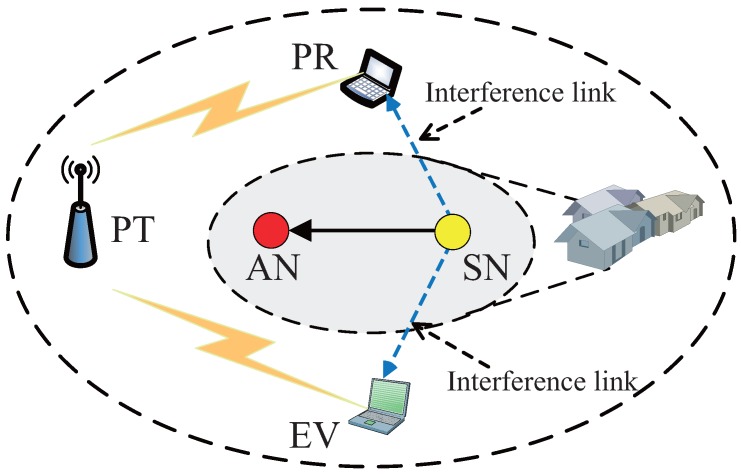
The system model of the proposed strategy.

In this system, a CRSN coexists with a primary network, as shown in Figure 1. The CRSN consists of a SN and a sink node (AN), in which the SN needs to periodically deliver its sensing information to the AN. The primary network consists of a primary transmit (PT) and a primary receiver (PR). In addition, there is an eavesdropper (EV), who eavesdrops on the primary confidential information. We assume that the eavesdropper is known to the primary network. This assumption is valid when the eavesdropper is a legitimate user that is untrusted by the primary network and can eavesdrop on the confidential messages or transmit and receive its own messages in the different time slots [31,32,33,34,35]. In this system, the CRSN lacks spectrum resources and needs to share the licensed spectrum for its data transmission through spectrum sensing. Due to the inevitable spectrum sensing errors, the SN’s transmission will interfere with the EV [36], and we can utilize the interference on the EV to protect the primary confidential messages. Therefore, in the proposed strategy, the confidential messages of the primary network can be protected, and as a reward, the primary network will permit limited spectrum sensing error probability.

We assume that the upper layer data packets are divided into equal frames, and the duration of each frame is *T*. Both the primary network and CRSN experience stationary, ergodic, independent and block Rayleigh fading [37], which indicates that the channel state will be invariant within a frame, but independently vary from one frame to another. The channel power gains of PT→PR, PT→EV, PT→SN, PT→AN, SN→PR, SN→EV and SN→AN are denoted as $g_{tr}$, $g_{te}$, $g_{ts}$, $g_{ta}$, $g_{sr}$, $g_{se}$ and $g_{sa}$, respectively, and they are assumed to follow exponential distributions with parameters $\sigma_{tr}^{2}$, $\sigma_{te}^{2}$, $\sigma_{ts}^{2}$, $\sigma_{ta}^{2}$, $\sigma_{sr}^{2}$, $\sigma_{se}^{2}$ and $\sigma_{sa}^{2}$, respectively. We assume that all noise variables in the system are cyclic symmetry complex Gaussian random variables with zero-mean and unit variance. The power budgets of the PT and SN are denoted as $P_{b}$ and $P_{s}$, respectively. To guarantee the security of the primary confidential messages, Wyner’s wiretap encoding scheme is adopted in this system, which indicates that the transmission rate and secrecy rate of the confidential messages are set as $R_{t}$ and $R_{sec}$, respectively. The difference between $R_{t}$ and $R_{sec}$, denoted as $R_{e}=R_{t}-R_{sec}$, is the information redundancy available against eavesdropping. A secrecy outage event occurs when the wiretap channel rate exceeds the information redundancy $R_{sec}$. We assume that the channel state information (CSI) associated with the primary network and CRSN can be perfectly estimated by the SN and PR, and this CSI information is reliably fed back to the AN and PT, respectively. In addition, since the eavesdropper is a known user, we assume that the CSI associated with the EV is available. This assumption is valid when the eavesdropper is an active user [38], a subscribed user [39], a jammer or a classical eavesdropper [40]. Even for the passive eavesdropper, it also can estimate the CSI through local oscillator power inadvertently leaked from the eavesdropper’s receiver radio frequency front-end [41].

The transmission activities of the primary network can be modeled as a binary-hypotheses problem [42]; the channel idle probability is denoted as $P\left(H_{0}\right)$, and the channel busy probability is denoted as $P\left(H_{1}\right)$, where $H_{0}$ denotes that the spectrum is idle and $H_{1}$ denotes that the spectrum is occupied. In this system, the SN firstly senses the licensed spectrum with the energy detection method during the first *τ* part of each frame [43]. The false alarm probability and the detection probability are given by:

(1) $$\begin{array}{c} \left\{\begin{array}{c} p_{f}=Q\left(\left(\frac{\varepsilon }{\sigma^{2}}-1\right)\sqrt{\tau f_{s}}\right),\\ p_{d}=Q\left(\left(\frac{\varepsilon }{\sigma^{2}}-\gamma -1\right)\sqrt{\frac{\tau f_{s}}{2\gamma +1}}\right) \end{array}\right. \end{array}$$

where $Q\left(x\right)=\frac{1}{2\pi }\int_{x}^{+\infty }e^{-\frac{t^{2}}{2}}dt$, *γ* is the received signal-to-noise ratio (SNR), $f_{s}$ is the sampling frequency, *ε* is the detection threshold and $\sigma^{2}=1$ is the noise variance.

According to the PT’s activities and spectrum sensing results, there are four system states, which are listed as below:

State0: the spectrum is idle and detected as idle;
State1: the spectrum is occupied and detected as idle;
State2: the spectrum is idle and detected as busy;
State3:the spectrum is occupied and detected as busy.

In State0 and State2, the spectrum is idle, and the SN can communicate with the AN freely. In State1 and State3, the SN should transmit with optimal power to protect the PT’s confidential messages. In return, the primary network tolerates limited spectrum sensing error probability. In the next section, we will carefully interpret the proposed strategy.

## 3. The Reciprocally-Benefited Secure Transmission Strategy

In this section, we firstly analyze the SN’s transmission rate and the primary secrecy rate for each state. Then, we optimally allocate the SN’s transmit power for each state, so that the average transmission rate of the SN is maximized under the constraint of the maximum permitted primary secrecy outage probability.

### 3.1. Problem Formulation

In State0, the SN can correctly detect the spectrum state as idle with probability:

(2) $$P_{0}=P\left(H_{0}\right)\left(1-p_{f}\right).$$

Then, the SN accesses the spectrum and transmits with rate $R_{s}^{\left(0\right)}$, which is given by:

(3) $$\begin{array}{c} R_{s}^{\left(0\right)}=\frac{T-\tau }{T}\log_{2}\left(1+g_{sa}p_{s}^{\left(0\right)}\right) \end{array}$$

where $p_{s}^{\left(0\right)}$ is the SN’s transmit power in this state. Since the PT stops its transmission, the secrecy rate of this state is zero.

In State1, the spectrum is occupied and incorrectly detected as idle with probability:

(4) $$P_{1}=P\left(H_{1}\right)\left(1-p_{d}\right).$$

Under the interference from the PT, the SN transmits with power $p_{s}^{\left(1\right)}$ and achieves rate $R_{s}^{\left(1\right)}$, which is given by:

(5) $$\begin{array}{c} R_{s}^{\left(1\right)}=\frac{T-\tau }{T}\log_{2}\left(1+\frac{g_{sa}p_{s}^{\left(1\right)}}{1+g_{ta}P_{b}}\right). \end{array}$$

The SN’s transmission will interfere with the EV, and the secrecy rate of the primary network is:

(6) $$\begin{array}{cc} R_{b}^{\left(1\right)}= & (\frac{\tau }{T}\left(\log_{2}\left(1+g_{tr}P_{b}\right)-\log_{2}\left(1+g_{te}P_{b}\right)\right)\\ & +{\left.\frac{T-\tau }{T}\left(\log_{2}\left(1+\frac{g_{tr}P_{b}}{1+g_{sr}p_{s}^{\left(1\right)}}\right)-\log_{2}\left(1+\frac{g_{te}P_{b}}{1+g_{se}p_{s}^{\left(1\right)}}\right)\right)\right)}^{+} \end{array}$$

where the first and second items in Equation (6) are the secrecy rates during the spectrum sensing phase and data transmission phase in State1, respectively.

In State2, the spectrum is idle and incorrectly detected as busy with probability:

(7) $$\begin{array}{c} P_{2}=P\left(H_{0}\right)p_{f}. \end{array}$$

In this state, the SN will transmit with power $p_{s}^{\left(2\right)}$ and achieve rate $R_{s}^{\left(2\right)}$, which is given by:

(8) $$\begin{array}{c} R_{s}^{\left(2\right)}=\frac{T-\tau }{T}\log_{2}\left(1+g_{sa}p_{s}^{\left(2\right)}\right). \end{array}$$

Since the PT stops its transmission, the secrecy rate of this state is zero.

In State3, the PT occupies the spectrum, and the SN detects the PT’s transmission with probability:

(9) $$P_{3}=P\left(H_{1}\right)p_{d}.$$

To protect the PT’s transmission, the SN transmits with power $p_{s}^{\left(3\right)}$ and achieves rate $R_{s}^{\left(3\right)}$, which is given by:

(10) $$\begin{array}{c} R_{s}^{\left(3\right)}=\frac{T-\tau }{T}\log_{2}\left(1+\frac{g_{sa}p_{s}^{\left(3\right)}}{1+g_{ta}P_{b}}\right). \end{array}$$

Then, under the interference from the SN, the secrecy rate of the primary network is:

(11) $$\begin{array}{cc} R_{b}^{\left(3\right)}= & (\frac{\tau }{T}\left(\log_{2}\left(1+g_{tr}P_{b}\right)-\log_{2}\left(1+g_{te}P_{b}\right)\right)\\ & +{\left.\frac{T-\tau }{T}\left(\log_{2}\left(1+\frac{g_{tr}P_{b}}{1+g_{sr}p_{s}^{\left(3\right)}}\right)-\log_{2}\left(1+\frac{g_{te}P_{b}}{1+g_{se}p_{s}^{\left(3\right)}}\right)\right)\right)}^{+}. \end{array}$$

where the first and second items are the secrecy rates during the spectrum sensing phase and data transmission phase in State3, respectively.

Therefore, the average transmission rate of the SN is given by:

(12) $$\begin{array}{cc} R_{s} & =E\left(R_{s}^{\left(0\right)}P_{0}+R_{s}^{\left(1\right)}P_{1}+R_{s}^{\left(2\right)}P_{2}+R_{s}^{\left(3\right)}P_{3}\right)\\ & =E\left(R_{s}^{\left(0\right)}\right)P_{0}+E\left(R_{s}^{\left(1\right)}\right)P_{1}+E\left(R_{s}^{\left(2\right)}\right)P_{2}+E\left(R_{s}^{\left(3\right)}\right)P_{3} \end{array}$$

where $E\left(\cdot \right)$ is the expectation operation. Under the SN’s interference, the secrecy outage probability of the primary network is:

(13) $$\begin{array}{c} P_{sec}^{out}=\mathrm{Pr}\left(R_{b}^{\left(1\right)}\leq R_{sec}\right)P_{1}+\mathrm{Pr}\left(R_{b}^{\left(3\right)}\leq R_{sec}\right)P_{3}. \end{array}$$

To maximize the SN’s average transmission rate under the constraint of the primary maximum permitted secrecy outage probability, the optimal power allocation problem is formulated as:

(14) $$\begin{array}{ccc} P1: & \max_{p_{s}^{\left(i\right)},\;i=0,1,2,3} & R_{s}\\ & s.t. & \left\{\begin{array}{c} P_{sec}^{out}\leq P_{th},\\ P_{sn}^{ave}\leq P_{av},\\ 0\leq p_{s}^{\left(i\right)}\leq P_{s},\;i=0,1,2,3 \end{array}\right. \end{array}$$

where $P_{th}$ is the maximum permitted primary secrecy outage probability, $P_{av}$ is the SN’s average power budget and $P_{sn}^{ave}$ is the SN’s average power consumption, which is given by:

(15) $$\begin{array}{c} P_{sn}^{ave}=\frac{T-\tau }{T}\left(E\left(p_{s}^{\left(0\right)}\right)P_{0}+E\left(p_{s}^{\left(1\right)}\right)P_{1}+E\left(p_{s}^{\left(2\right)}\right)P_{2}+E\left(p_{s}^{\left(3\right)}\right)P_{3}\right). \end{array}$$

### 3.2. Optimal Power Allocation

In this section, we will solve the optimization problem given in Equation (14) to acquire the optimal power allocation for each system state. Since the secrecy outage probability constraint in Equation (14) is not convex over $\left\{p_{s}^{\left(i\right)},\;i=0,1,2,3\right\}$, **P1** cannot be solved through the traditional convex optimization algorithms. To solve this problem, indicator functions $\varphi \left(p_{s}^{\left(1\right)}\right)$ and $\varphi \left(p_{s}^{\left(3\right)}\right)$ are introduced, which are given by:

(16) $$\begin{array}{c} \varphi \left(p_{s}^{\left(1\right)}\right)=\left\{\begin{array}{c} 0,\;R_{b}^{\left(1\right)}\geq R_{c},\\ 1,\;R_{b}^{\left(1\right)}<R_{c} \end{array}\right. \end{array}$$

and:

(17) $$\begin{array}{c} \varphi \left(p_{s}^{\left(3\right)}\right)=\left\{\begin{array}{c} 0,\;R_{b}^{\left(3\right)}\geq R_{sec},\\ 1,\;R_{b}^{\left(3\right)}<R_{sec} \end{array}\right. \end{array}$$

respectively. Since the spectrum sensing time is very short compared with the duration of a frame, we ignore the first items of the right side of $R_{b}^{\left(1\right)}$ and $R_{b}^{\left(3\right)}$ [44,45]. Then, ${\overset{ˇ}{R}}_{b}^{\left(1\right)}\approx R_{b}^{\left(1\right)}$ and ${\overset{ˇ}{R}}_{b}^{\left(3\right)}\approx R_{b}^{\left(3\right)}$ where ${\overset{ˇ}{R}}_{b}^{\left(1\right)}$ and ${\overset{ˇ}{R}}_{b}^{\left(3\right)}$ are given by:

(18) $$\begin{array}{c} \left\{\begin{array}{c} {\overset{ˇ}{R}}_{b}^{\left(1\right)}=\frac{T-\tau }{T}{\left(\log_{2}\left(1+\frac{g_{tr}P_{b}}{1+g_{sr}p_{s}^{\left(1\right)}}\right)-\log_{2}\left(1+\frac{g_{te}P_{b}}{1+g_{se}p_{s}^{\left(1\right)}}\right)\right)}^{+},\\ {\overset{ˇ}{R}}_{b}^{\left(3\right)}=\frac{T-\tau }{T}{\left(\log_{2}\left(1+\frac{g_{tr}P_{b}}{1+g_{sr}p_{s}^{\left(3\right)}}\right)-\log_{2}\left(1+\frac{g_{te}P_{b}}{1+g_{se}p_{s}^{\left(3\right)}}\right)\right)}^{+} \end{array}\right. \end{array}$$

respectively. Therefore, the optimal problem P1 can be rewritten as:

(19) $$\begin{array}{ccc} P2: & \max_{\begin{array}{c} p_{s}^{\left(i\right)},\\ i=0,1,2,3 \end{array}} & R_{s}\\ & s.t. & \left\{\begin{array}{c} E\left(\varphi \left(p_{s}^{\left(1\right)}\right)\right)P_{1}+E\left(\varphi \left(p_{s}^{\left(3\right)}\right)\right)P_{3}\leq P_{th},\\ P_{sn}^{ave}\leq P_{av},\\ 0\leq p_{s}^{\left(i\right)}\leq P_{s},\;i=0,1,2,3. \end{array}\right. \end{array}$$

The optimal problem P2 can be solved through the Lagrange dual method [46]. The Lagrange function of P2 can be derived as:

(20) $$\begin{array}{c} L\left(p_{s}^{\left(1\right)},p_{s}^{\left(1\right)},p_{s}^{\left(2\right)},p_{s}^{\left(3\right)}\right)=R_{s}+\mu \left(P_{th}-\left(\varphi \left(p_{s}^{\left(1\right)}\right)\right)P_{1}-\left(\varphi \left(p_{s}^{\left(3\right)}\right)\right)P_{3}\right)+\lambda \left(P_{av}-P_{sn}^{ave}\right). \end{array}$$

Then, the Lagrange dual function of the maximum problem, denoted by $G\left(\lambda ,\mu \right)$, can be formulated as:

(21) $$\begin{array}{ccc} P3: & \max_{\begin{array}{c} p_{s}^{\left(i\right)},\\ i=0,1,2,3 \end{array}} & L\left(p_{s}^{\left(0\right)},p_{s}^{\left(1\right)},p_{s}^{\left(2\right)},p_{s}^{\left(3\right)}\right)\\ & s.t. & 0\leq p_{s}^{\left(i\right)}\leq P_{s},\;i=0,1,2,3. \end{array}$$

The dual problem is:

(22) $$\begin{array}{c} P4:\;\min_{\lambda ,\mu }\;G\left(\lambda ,\mu \right)\;s.t.\;\lambda ,\mu >0. \end{array}$$

Since the optimal variables of $p_{s}^{\left(0\right)}$, $p_{s}^{\left(1\right)}$, $p_{s}^{\left(2\right)}$, $p_{s}^{\left(3\right)}$ corresponding to the four system states are independent of each other, the Lagrange function can be written as:

(23) $$\begin{array}{cc} L\left(p_{s}^{\left(1\right)},p_{s}^{\left(1\right)},p_{s}^{\left(2\right)},p_{s}^{\left(3\right)}\right) & =E\left(R_{s}^{\left(0\right)}\right)P_{0}+\lambda \left(P_{av}-\frac{T-\tau }{T}E\left(p_{s}^{\left(0\right)}\right)P_{0}\right)\\ & +E\left(R_{s}^{\left(1\right)}\right)P_{1}+\mu \left(P_{th}-\varphi \left(p_{s}^{\left(1\right)}\right)P_{1}\right)+\lambda \left(P_{av}-\frac{T-\tau }{T}E\left(p_{s}^{\left(1\right)}\right)P_{1}\right)\\ & +E\left(R_{s}^{\left(2\right)}\right)P_{2}+\lambda \left(P_{av}-\frac{T-\tau }{T}E\left(p_{s}^{\left(2\right)}\right)P_{2}\right)\\ & +E\left(R_{s}^{\left(3\right)}\right)P_{3}+\mu \left(P_{th}-\varphi \left(p_{s}^{\left(3\right)}\right)\right)P_{3}+\lambda \left(P_{av}-\frac{T-\tau }{T}E\left(p_{s}^{\left(3\right)}\right)P_{3}\right)\\ & -\mu P_{th}-3\lambda P_{av} \end{array}$$

Then, the problem **P3** can be decomposed as:

(24) $$\begin{array}{cc} P3a: & \max_{p_{s}^{\left(0\right)}}\;E\left(R_{s}^{\left(0\right)}\right)P_{0}+\lambda \left(P_{av}-\frac{T-\tau }{T}E\left(p_{s}^{\left(0\right)}\right)P_{0}\right)\\ & s.t.\;0\leq p_{s}^{\left(0\right)}\leq P_{s}, \end{array}$$

(25) $$\begin{array}{cc} P3b: & \max_{p_{s}^{\left(1\right)}}\;E\left(R_{s}^{\left(1\right)}\right)P_{1}+\mu \left(P_{th}-\varphi \left(p_{s}^{\left(1\right)}\right)P_{1}\right)+\lambda \left(P_{av}-\frac{T-\tau }{T}E\left(p_{s}^{\left(1\right)}\right)P_{1}\right)\\ & s.t.\;0\leq p_{s}^{\left(1\right)}\leq P_{s}, \end{array}$$

(26) $$\begin{array}{cc} P3c: & \max_{p_{s}^{\left(2\right)}}\;E\left(R_{s}^{\left(2\right)}\right)P_{2}+\lambda \left(P_{av}-\frac{T-\tau }{T}E\left(p_{s}^{\left(2\right)}\right)P_{2}\right)\\ & s.t.\;0\leq p_{s}^{\left(2\right)}\leq P_{s} \end{array}$$

and:

(27) $$\begin{array}{cc} P3d: & \max_{p_{s}^{\left(3\right)}}\;E\left(R_{s}^{\left(3\right)}\right)P_{3}+\mu \left(P_{th}-\varphi \left(p_{s}^{\left(3\right)}\right)P_{3}\right)+\lambda \left(P_{av}-\frac{T-\tau }{T}E\left(p_{s}^{\left(3\right)}\right)P_{3}\right)\\ & s.t.\;0\leq p_{s}^{\left(3\right)}\leq P_{s}. \end{array}$$

Therefore, the problem can be decomposed into four optimal problems, corresponding to the transmit power $p_{s}^{\left(0\right)}$, $p_{s}^{\left(1\right)}$, $p_{s}^{\left(2\right)}$, $p_{s}^{\left(3\right)}$, respectively.

For State0 and State2, we firstly derive the partial derivatives of $p_{s}^{\left(0\right)}$ and $p_{s}^{\left(2\right)}$ as:

(28) $$\begin{array}{c} \left\{\begin{array}{c} \frac{\partial L}{\partial p_{s}^{\left(0\right)}}=\frac{\left(T-\tau \right)g_{sa}}{T\left(1+g_{sa}p_{s}^{\left(0\right)}\right)}P_{0}-\lambda P_{0}\frac{T-\tau }{T},\\ \frac{\partial L}{\partial p_{s}^{\left(2\right)}}=\frac{\left(T-\tau \right)g_{sa}}{T\left(1+g_{sa}p_{s}^{\left(2\right)}\right)}P_{2}-\lambda P_{2}\frac{T-\tau }{T}. \end{array}\right. \end{array}$$

Applying the Karush–Kuhn–Tucker conditions and setting the partial derivatives equal to zero, we can acquire the SN’s transmit power in State0 and State2 as:

(29) $$\begin{array}{c} \left\{\begin{array}{c} p_{s}^{\left(0\right)}={\left(\frac{1}{\lambda }-\frac{1}{g_{sa}}\right)}^{+},\\ p_{s}^{\left(2\right)}={\left(\frac{1}{\lambda }-\frac{1}{g_{sa}}\right)}^{+}. \end{array}\right. \end{array}$$

For $p_{s}^{\left(1\right)}$ in State1, the decomposed optimal problem is:

(30) $$\begin{array}{c} P5:\;\max_{p_{s}^{\left(1\right)}}\;R_{s}^{\left(1\right)}P_{1}+\lambda \left(P_{av}-\frac{T-\tau }{T}P_{1}p_{s}^{\left(1\right)}\right)+\mu \left(P_{th}-P_{1}\varphi \left(p_{s}^{\left(1\right)}\right)\right). \end{array}$$

Since the value $\varphi \left(p_{s}^{\left(1\right)}\right)$ is determined by ${\overset{ˇ}{R}}_{b}^{\left(1\right)}$ and $R_{sec}$, the optimal $p_{s}^{\left(1\right)}$ is derived according to the relationship between ${\overset{ˇ}{R}}_{b}^{\left(1\right)}$ and $R_{sec}$. When ${\overset{ˇ}{R}}_{b}^{\left(1\right)}=R_{sec}$, we can acquire the following equation as:

(31) $$A{\left(p_{s}^{\left(1\right)}\right)}^{2}+Bp_{s}^{\left(1\right)}+C=0.$$

where $\nu =2^{\frac{R_{sec}\left(T-\tau \right)}{T}}$ and:

(32) $$\begin{array}{c} \left\{\begin{array}{c} A=\left(\nu -1\right)g_{sr}g_{se},\\ B=\left(\nu -1\right)\left(g_{sr}+g_{se}\right)+\left(\nu g_{sr}g_{te}-g_{se}g_{tr}\right)P_{b},\\ C=\nu \left(1+g_{te}P_{b}\right)-\left(1+g_{tr}P_{b}\right). \end{array}\right. \end{array}$$

**Remark** **1.** *In the derivation of Equation (18), we assume that ${\overset{ˇ}{R}}_{b}^{\left(1\right)}\approx R_{b}^{\left(1\right)}$ and ${\overset{ˇ}{R}}_{b}^{\left(3\right)}\approx R_{b}^{\left(3\right)}$. Compared with $R_{b}^{\left(1\right)}$ and $R_{b}^{\left(3\right)}$, the term $\frac{\tau }{T}\left(\log_{2}\left(1+g_{tr}P_{b}\right)-\log_{2}\left(1+g_{te}P_{b}\right)\right)$ in $R_{b}^{\left(1\right)}$ and $R_{b}^{\left(3\right)}$ is ignored. Next, we will evaluate the effect of this assumption and take State1 for example. When the information can be securely and reliably transmitted to the primary destination, the effect of this assumption can be evaluated by:*

(33) $$\begin{array}{cc} \theta^{\left(1\right)} & =\frac{\frac{\tau }{T}\left(\log_{2}\left(1+g_{tr}P_{b}\right)-\log_{2}\left(1+\frac{g_{tr}P_{b}}{1+g_{sr}p_{s}^{\left(1\right)}}\right)\right)}{R_{b}^{\left(1\right)}}\\ & =\frac{1}{1+\frac{T-\tau }{\tau }\times \frac{\left(\log_{2}\left(1+\frac{g_{tr}P_{b}}{1+g_{sr}p_{s}^{\left(1\right)}}\right)-\log_{2}\left(1+\frac{g_{te}P_{b}}{1+g_{se}p_{s}^{\left(1\right)}}\right)\right)}{\left(\log_{2}\left(1+g_{tr}P_{b}\right)-\log_{2}\left(1+g_{te}P_{b}\right)\right)}}\\ & \overset{\left(a\right)}{\leq }\frac{1}{1+\frac{T-\tau }{\tau }\times \frac{R_{sec}}{\log_{2}\left(1+g_{tr}P_{b}\right)}} \end{array}$$

*where in $\left(a\right)$, we utilize the approximations of:*

(34) $$\begin{array}{c} \left\{\begin{array}{c} \log_{2}\left(1+\frac{g_{tr}P_{b}}{1+g_{sr}p_{s}^{\left(1\right)}}\right)-\log_{2}\left(1+\frac{g_{te}P_{b}}{1+g_{se}p_{s}^{\left(1\right)}}\right)\geq R_{sec},\\ \log_{2}\left(1+g_{tr}P_{b}\right)-\log_{2}\left(1+g_{te}P_{b}\right)\leq \log_{2}\left(1+g_{tr}P_{b}\right). \end{array}\right. \end{array}$$

*Since the information can be securely and reliably transmitted to the primary destination, $\log_{2}\left(1+g_{tr}P_{b}\right)\geq R_{t}>R_{sec}$. Therefore,*

(35) $$\begin{array}{c} \theta^{\left(1\right)}<\frac{\tau }{T}. \end{array}$$

In addition, as the spectrum sensing time is too short compared with the data transmission time and can be ignored [44,45], therefore $\theta^{\left(1\right)}$ is very small, and we can ignore the term $\frac{\tau }{T}\left(\log_{2}\left(1+g_{tr}P_{b}\right)-\log_{2}\left(1+g_{te}P_{b}\right)\right)$ in $R_{b}^{\left(1\right)}$. Similarly, the term $\frac{\tau }{T}\left(\log_{2}\left(1+g_{tr}P_{b}\right)-\log_{2}\left(1+g_{te}P_{b}\right)\right)$ in $R_{b}^{\left(3\right)}$ can be ignored.

#### 3.2.1. Equation (31) Has No Root: $B^{2}-4AC<0$

For this scenario, ${\overset{ˇ}{R}}_{b}^{\left(1\right)}$ is always less than $R_{sec}$, which means that the primary network will experience secrecy outage even under the assistance of CRSN. Then, the optimal problem can be rewritten as:

(36) $$\begin{array}{c} P6:\;\max_{p_{s}^{\left(1\right)}}\;R_{s}^{\left(1\right)}P_{1}+\lambda \left(P_{av}-\frac{T-\tau }{T}P_{1}p_{s}^{\left(1\right)}\right)+\mu \left(P_{th}-P_{1}\right). \end{array}$$

Set $l\left(p_{s}^{\left(1\right)}\right)=R_{s}^{\left(1\right)}P_{1}+\lambda \left(P_{av}-\frac{T-\tau }{T}P_{1}p_{s}^{\left(1\right)}\right)+\mu \left(P_{th}-P_{1}\right).$ According to the Karush–Kuhn–Tucker conditions, we can derive:

(37) $$\begin{array}{c} \left\{\begin{array}{c} \frac{dl}{dp_{s}^{\left(1\right)}}<0,\;\mathrm{if}\;p_{s}^{\left(1\right)}=0,\\ \frac{dl}{dp_{s}^{\left(1\right)}}=0,\;\mathrm{if}\;0\leq p_{s}^{\left(1\right)}\leq P_{s},\\ \frac{dl}{dp_{s}^{\left(1\right)}}>0,\;\mathrm{if}\;p_{s}^{\left(1\right)}=P_{s}. \end{array}\right. \end{array}$$

Therefore, the optimal $p_{s}^{\left(1\right)}$ can be acquired as:

(38) $$\begin{array}{c} p_{s}^{\left(1\right)}=\left\{\begin{array}{cc} 0, & \frac{1}{\lambda }<\frac{1+g_{ta}P_{p}}{g_{sa}},\\ \frac{1}{\lambda }-\frac{1+g_{ta}P_{b}}{g_{sa}}, & \frac{1+g_{ta}P_{b}}{g_{sa}}\leq \frac{1}{\lambda }\leq P_{s}+\frac{1+g_{ta}P_{b}}{g_{sa}},\\ P_{s}, & \frac{1}{\lambda }>P_{s}+\frac{1+g_{ta}P_{b}}{g_{sa}}. \end{array}\right. \end{array}$$

#### 3.2.2. Equation (31) Has Two Roots: $B^{2}-4AC\geq 0$

For this scenario, Equation (31) has two roots denoted as $p_{s}^{\left(1\right)-}$ and $p_{s}^{\left(1\right)+}$. Let the first-order derivative of the optimal problem P6 equal zero; the optimal $p_{s}^{\left(1\right)*}$ can be derived as:

(39) $$p_{s}^{\left(1\right)*}=\frac{1}{\lambda }-\frac{1+g_{ta}P_{b}}{g_{sa}}.$$

For this scenario, the optimal transmit power of $p_{s}^{\left(1\right)}$ is derived as:

- $p_{s}^{\left(1\right)}=0$, when $p_{s}^{\left(1\right)-}\leq p_{s}^{\left(1\right)*}\leq 0\leq p_{s}^{\left(1\right)+}<P_{s}$, $p_{s}^{\left(1\right)-}<p_{s}^{\left(1\right)*}\leq 0\leq P_{s}\leq p_{s}^{\left(1\right)+}$, $p_{s}^{\left(1\right)-}<p_{s}^{\left(1\right)+}\leq p_{s}^{\left(1\right)*}\leq 0<P_{s}$ and $p_{s}^{\left(1\right)-}\leq p_{s}^{\left(1\right)*}<p_{s}^{\left(1\right)+}\leq 0<P_{s}$.
- $p_{s}^{\left(1\right)}=p_{s}^{\left(1\right)-}$, when $0<p_{s}^{\left(1\right)*}\leq p_{s}^{\left(1\right)-}\leq P_{pk}<p_{s}^{\left(1\right)+}\;and\;f\left(p_{s}^{\left(1\right)*}\right)\leq f\left(p_{s}^{\left(1\right)-}\right)$ and $0<p_{s}^{\left(1\right)*}\leq p_{s}^{\left(1\right)-}<p_{s}^{\left(1\right)+}\leq P_{s}\;and\;f\left(p_{s}^{\left(1\right)-}\right)\leq f\left(p_{s}^{\left(1\right)*}\right)$.
- $p_{s}^{\left(1\right)}=p_{s}^{\left(1\right)*}$, when $p_{s}^{\left(1\right)-}\leq 0\leq p_{s}^{\left(1\right)+}<p_{s}^{\left(1\right)*}\leq P_{s}\;and\;f\left(p_{s}^{\left(1\right)+}\right)\leq f\left(p_{s}^{\left(1\right)*}\right)$, $p_{s}^{\left(1\right)-}\leq 0<p_{s}^{\left(1\right)*}\leq p_{s}^{\left(1\right)+}<P_{s}$, $p_{s}^{\left(1\right)-}\leq 0<p_{s}^{\left(1\right)*}\leq P_{s}\leq p_{s}^{\left(1\right)+}$, $0\leq p_{s}^{\left(1\right)-}<p_{s}^{\left(1\right)*}\leq P_{s}<p_{s}^{\left(1\right)+}$, $0<p_{s}^{\left(1\right)*}\leq p_{s}^{\left(1\right)-}\leq P_{s}<p_{s}^{\left(1\right)+}\;and\;f\left(p_{s}^{\left(1\right)*}\right)>f\left(p_{s}^{\left(1\right)-}\right)$, $0\leq p_{s}^{\left(1\right)-}<p_{s}^{\left(1\right)+}<p_{s}^{\left(1\right)*}\leq P_{s}\;and\;f\left(p_{s}^{\left(1\right)+}\right)\leq f\left(p_{s}^{\left(1\right)*}\right)$, $0\leq p_{s}^{\left(1\right)-}<p_{s}^{\left(1\right)*}\leq p_{s}^{\left(1\right)+}\leq P_{s}$, $0<p_{s}^{\left(1\right)*}\leq p_{s}^{\left(1\right)-}<p_{s}^{\left(1\right)+}\leq P_{s}\;and\;f\left(p_{s}^{\left(1\right)-}\right)>f\left(p_{s}^{\left(1\right)*}\right)$ and $p_{s}^{\left(1\right)-}<p_{s}^{\left(1\right)+}\leq 0<p_{s}^{\left(1\right)*}\leq P_{s}$.
- $p_{s}^{\left(1\right)}=p_{s}^{\left(1\right)+}$, when $p_{s}^{\left(1\right)-}\leq 0\leq p_{s}^{\left(1\right)+}<P_{s}<p_{s}^{\left(1\right)*}\;and\;f\left(p_{s}^{\left(1\right)+}\right)>f\left(P_{s}\right)$, $p_{s}^{\left(1\right)-}\leq 0\leq p_{s}^{\left(1\right)+}<p_{s}^{\left(1\right)*}\leq P_{s}\;and\;f\left(p_{s}^{\left(1\right)+}\right)>f\left(p_{s}^{\left(1\right)*}\right)$, $0\leq p_{s}^{\left(1\right)-}<p_{s}^{\left(1\right)+}\leq P_{s}<p_{s}^{\left(1\right)*}\;and\;f\left(p_{s}^{\left(1\right)+}\right)>f\left(P_{s}\right)$ and $0\leq p_{s}^{\left(1\right)-}<p_{s}^{\left(1\right)+}<p_{s}^{\left(1\right)*}\leq P_{s}\;and\;f\left(p_{s}^{\left(1\right)+}\right)>f\left(p_{s}^{\left(1\right)*}\right)$.
- $p_{s}^{\left(1\right)}=P_{s}$, when $p_{s}^{\left(1\right)-}\leq 0\leq p_{s}^{\left(1\right)+}<P_{s}<p_{s}^{\left(1\right)*}\;and\;f\left(p_{s}^{\left(1\right)+}\right)\leq f\left(P_{s}\right)$, $p_{s}^{\left(1\right)-}\leq 0\leq p_{s}^{\left(1\right)+}<P_{s}<p_{s}^{\left(1\right)*}\;and\;f\left(p_{s}^{\left(1\right)+}\right)\leq f\left(P_{s}\right)$, $p_{s}^{\left(1\right)-}\leq 0\leq P_{s}<p_{s}^{\left(1\right)*}\leq p_{s}^{\left(1\right)+}$, $p_{s}^{\left(1\right)-}\leq 0\leq P_{s}<p_{s}^{\left(1\right)*}\leq p_{s}^{\left(1\right)+}$, $0\leq p_{s}^{\left(1\right)-}\leq P_{s}<p_{s}^{\left(1\right)+}<p_{s}^{\left(1\right)*}$, $0\leq p_{s}^{\left(1\right)-}\leq P_{s}<p_{s}^{\left(1\right)*}\leq p_{s}^{\left(1\right)+}$, $0\leq p_{s}^{\left(1\right)-}<p_{s}^{\left(1\right)+}\leq P_{s}<p_{s}^{\left(1\right)*}\;and\;f\left(p_{s}^{\left(1\right)+}\right)\leq f\left(P_{s}\right)$, $p_{s}^{\left(1\right)-}<p_{s}^{\left(1\right)+}\leq 0<P_{s}<p_{s}^{\left(1\right)*}$, $0<P_{s}\leq p_{s}^{\left(1\right)-}<p_{s}^{\left(1\right)+}<p_{s}^{\left(1\right)*}$, $0<P_{s}\leq p_{s}^{\left(1\right)-}<p_{s}^{\left(1\right)*}\leq p_{s}^{\left(1\right)+}$, $0<P_{s}<p_{s}^{\left(1\right)*}\leq p_{s}^{\left(1\right)-}<p_{s}^{\left(1\right)+}$ and $0\leq p_{s}^{\left(1\right)*}\leq P_{s}\leq p_{s}^{\left(1\right)-}<p_{s}^{\left(1\right)+}$.

**Proof.**  The derivation process of $p_{s}^{\left(1\right)}$ is shown in Appendix A.  ☐

Similarly, $p_{s}^{\left(3\right)}$ is derived with the same method as $p_{s}^{\left(1\right)}$, which is omitted due to the space limitation. Then, the optimal transmit power for four system states is derived by taking into account the SN’s average power constraint, spectrum sensing errors, as well as the primary secure requirement. Next, we will briefly analyze the performance of the proposed strategy.

## 4. Performance Analysis

In this section, we will give a further discussion for the power allocation strategy. In addition, the performance tradeoff between the transmission rate of CRSN and the secrecy outage probability of the primary network is investigated. Then, we investigate the primary secrecy rate for the asymptotic scenarios and derive the closed-form expression of the SN’s transmission outage probability.

### 4.1. Further Discussion of the SN’s Transmit Power

In Section 3.2, we have derived the optimal transmit power for four system states. Now, some further discussions about the power allocation strategy will be given in this section.

- In State0 and State2, the channel is idle, and the SN will access the licensed spectrum with power $p_{s}^{\left(0\right)}$ and $p_{s}^{\left(2\right)}$, respectively. Under the average transmit power constraint, $p_{s}^{\left(0\right)}$ and $p_{s}^{\left(2\right)}$ are derived through the optimal water-filling method.
- In State1 and State3, the SN’s transmission will affect the secrecy outage probability of the primary network. Since the derivation of $p_{s}^{\left(1\right)}$ is similar with $p_{s}^{\left(3\right)}$, we take $p_{s}^{\left(1\right)}$ for example.
  (1) If $B^{2}-4AC<0$, the channel quality between the PT and the PR is always worse than the channel quality between the PT and the EV. Under this condition, the primary information security cannot be guaranteed even though the SN optimally allocates its transmit power to interfere with the EV. Therefore, for this case, the SN will access the licensed spectrum with its maximum transmission power as shown in Equation (38).
  (2) If $B^{2}-4AC\geq 0$, there are six cases, which are shown in Appendix A, and we take $p_{s}^{\left(1\right)-}\leq 0<p_{s}^{\left(1\right)+}\leq P_{s}$ and $p_{s}^{\left(1\right)-}\leq 0<p_{s}^{\left(1\right)+}\leq P_{s}$ for example.
    Case1: If $p_{s}^{\left(1\right)-}\leq 0<P_{s}\leq p_{s}^{\left(1\right)+}$, it indicates that the direct transmission channel quality of the primary network is sufficiently good, and the eavesdropper cannot acquire the primary confidential messages even without the assistance of the SN. In this case, the SN will transmit with its maximum power as shown in Equation (38).
    Case2: If $p_{s}^{\left(1\right)-}\leq 0<p_{s}^{\left(1\right)+}\leq P_{s}$, it indicates that the channel quality between the PT and the PR is neither sufficiently better nor worse than the channel quality between the PT and the EV. In this scenario, the tradeoff between the CRSN’s performance and the primary secrecy outage probability constraint needs to be studied. Set $h\left(p_{s}^{\left(1\right)}\right)=R_{s}^{\left(1\right)}P_{1}+\lambda \left(P_{av}-\frac{T-\tau }{T}P_{1}p_{s}^{\left(1\right)}\right)+\mu P_{th}$ and $f\left(p_{s}^{\left(1\right)}\right)=h\left(p_{s}^{\left(1\right)}\right)-\mu \varphi \left(p_{s}^{\left(1\right)}\right)$. Suppose $\ell =\min\left\{p_{s}^{\left(1\right)*},P_{s}\right\}$. Obviously, if the CRSN refuses the cooperation request from the PT, the CRSN can acquire the maximum transmission rate. We can denote *μ* as the cost that the CRSN has to pay if the secrecy outage probability of the primary network is caused. If $f\left(\ell \right)+\mu <f\left(p_{s}^{\left(1\right)*}\right)$, the CRSN will transmit with a large power *ℓ* to maximize its own performance and, thus, causes a primary secrecy outage because the CRSN only needs to pay a relatively small cost. However, if $f\left(\ell \right)+\mu \geq f\left(p_{s}^{\left(1\right)*}\right)$, the CRSN will use a low power $p_{s}^{\left(1\right)*}$ to guarantee the secure transmission of the primary network since the cost due to the secrecy outage is large. Therefore, the performance tradeoff between the transmission rate of the CRSN and the secrecy outage probability of primary network can be acquired.

### 4.2. Asymptotic Secrecy Rate Analysis

In this section, we will give the asymptotic analysis for the primary secrecy rate under the condition that $P_{b}\to 0$ and $P_{s}\to \infty$. In State1 and State3, the secure capacities of the primary network are:

(40) $$\begin{array}{cc} R_{b}^{\left(i\right)} & \approx \frac{T-\tau }{T}{\left(\log_{2}\left(1+\frac{g_{tr}P_{b}}{1+g_{sr}p_{s}^{\left(i\right)}}\right)-\log_{2}\left(1+\frac{g_{te}P_{b}}{1+g_{se}p_{s}^{\left(1\right)}}\right)\right)}^{+}\\ & =\frac{T-\tau }{T}{\left(\log_{2}\left(\frac{1+g_{se}p_{s}^{\left(i\right)}}{1+g_{sr}p_{s}^{\left(i\right)}}\times \frac{1+g_{sr}p_{s}^{\left(i\right)}+g_{bm}P_{b}}{1+g_{se}p_{s}^{\left(i\right)}+g_{te}P_{b}}\right)\right)}^{+}, \end{array}$$

where i=1 and 3 stand for State1 and State3, respectively.

#### 4.2.1. $P_{b}\to 0$

When $P_{b}\to 0$, the secrecy rate of the primary network can be approximately quantified by its first-order derivative with respect to $P_{b}$ at $P_{b}=0$. Then, the secrecy rate can be acquired as:

(41) $$\begin{array}{c} R_{sec}^{\left(i\right)}\left(P_{b}\right)={\dot{R}}_{sec}^{\left(i\right)}\left(0\right)P_{b}+o\left(P_{b}\right),\;i=1,3, \end{array}$$

where ${\dot{R}}_{sec}^{\left(i\right)}\left(0\right)$ is the first-order derivative at $P_{b}=0$ and $o\left(P_{b}\right)$ denotes the high-order item. According to $R_{sec}^{\left(i\right)}$ in Section 3.1, the secrecy rate of the primary network can be rewritten as:

(42) $$\begin{array}{c} R_{sec}^{\left(i\right)}=\frac{T-\tau }{T}{\left(\frac{g_{bm}\left(1+g_{se}p_{s}^{\left(i\right)}\right)-g_{te}\left(1+g_{sr}p_{s}^{\left(i\right)}\right)}{\left(1+g_{sr}p_{s}^{\left(i\right)}\right)\left(1+g_{se}p_{s}^{\left(i\right)}\right)}P_{b}+o\left(P_{b}\right)\right)}^{+}, \end{array}$$

where the first item of Equation (42) is the first derivative of $R_{sec}^{\left(i\right)}$. Under this condition, the secrecy rate of the primary network is almost zero. The channel quality between the EV and the primary network and between the EV and the CRSN and the power allocation strategy have almost no effect on the secrecy rate.

#### 4.2.2. $P_{s}\to \infty$

When $P_{s}\to \infty$, the average transmit power constraint in P1 can be removed. Then, the optimal problem can be rewritten as:

(43) $$\begin{array}{c} P6:\max_{\begin{array}{c} p_{s}^{\left(i\right)},\\ i=0,1,2,3 \end{array}}R_{s}\;s.t.\;P_{sec}^{out}\leq P_{th}. \end{array}$$

Adopting the same method in Section 3.2, in State0 and State1, we can acquire:

(44) $$p_{s}^{\left(0\right)}=p_{s}^{\left(2\right)}=P_{s}.$$

In State1 and State3, the optimal power is:

- $B^{2}-4AC<0$, $p_{s}^{\left(1\right)}=p_{s}^{\left(3\right)}=P_{s}$.
- $B^{2}-4AC\geq 0$, there are two roots, which are denoted as $p_{s}^{\left(1\right)-}$ and $p_{s}^{\left(1\right)+}$. Set i=1,3. Since $R_{s}^{\left(i\right)}$ is a monotonically-increasing function with respect to $p_{s}^{\left(i\right)}$ and $P_{s}>p_{s}^{\left(1\right)+}$, $R_{s}^{\left(i\right)}\left(P_{s}\right)>\max\left\{R_{s}^{\left(i\right)}\left(p_{s}^{\left(1\right)-}\right),R_{s}^{\left(i\right)}\left(p_{s}^{\left(1\right)+}\right)\right\}$. Then, if $\max\left\{p_{s}^{\left(1\right)-},p_{s}^{\left(1\right)+}\right\}>0$, the optimal power is one value of zero, $p_{s}^{\left(i\right)-}$, $p_{s}^{\left(i\right)+}$ and $P_{s}$ that can maximize the function $\left(R_{s}^{\left(i\right)}-\mu \varphi \left(p_{s}^{\left(i\right)}\right)\right)$. Otherwise, the optimal power is $p_{s}^{\left(i\right)}=P_{s}$.

Then, substituting $p_{s}^{\left(1\right)}$ and $p_{s}^{\left(3\right)}$ into Equation (40), we can derive the secrecy rate of the primary network.

### 4.3. The Outage Probability Analysis of the CRSN

To guarantee the secure transmission of the primary network, the CRSN should dynamically adjust its transmission power. Set the target transmission rate of the SN as $R_{s}$. In State0 and State2, the SN will transmit without the interference from primary network and acquire the transmission rate as $R_{s}^{\left(0\right)}$ and $R_{s}^{\left(0\right)}$, respectively. Set the target transmission rate of the CRSN as $R_{s}$. Then, the outage probabilities for State0 and State2 are:

(45) $$\begin{array}{c} \left\{\begin{array}{c} P_{out}^{\left(0\right)}=\mathrm{Pr}\left(R_{s}^{\left(0\right)}<R_{s}\right)=1-\exp\left(-\frac{2^{\frac{TR_{s}}{T-\tau }}-1}{\sigma_{sh}^{2}p_{s}^{\left(0\right)}}\right),\\ P_{out}^{\left(2\right)}=\mathrm{Pr}\left(R_{s}^{\left(2\right)}<R_{s}\right)=1-\exp\left(-\frac{2^{\frac{TR_{s}}{T-\tau }}-1}{\sigma_{sh}^{2}p_{s}^{\left(2\right)}}\right), \end{array}\right. \end{array}$$

respectively. In State1 and State3, the SN’s transmission will be interfered by the primary network. Then, the outage probabilities corresponding to State1 and State3 are derived as:

(46) $$\begin{array}{c} \left\{\begin{array}{c} P_{out}^{\left(1\right)}=\mathrm{Pr}\left(R_{s}^{\left(1\right)}<R_{s}\right)=1-\frac{\sigma_{bh}^{2}\sigma_{sh}^{2}p_{s}^{\left(1\right)}}{\left(2^{\frac{TR_{s}}{T-\tau }}-1\right)\sigma_{bh}^{2}+\sigma_{sh}^{2}p_{s}^{\left(1\right)}}\exp\left(-\frac{2^{\frac{TR_{s}}{T-\tau }}-1}{\sigma_{sh}^{2}p_{s}^{\left(1\right)}}\right),\\ P_{out}^{\left(3\right)}=\mathrm{Pr}\left(R_{s}^{\left(1\right)}<R_{s}\right)=1-\frac{\sigma_{bh}^{2}\sigma_{sh}^{2}p_{s}^{\left(3\right)}}{\left(2^{\frac{TR_{s}}{T-\tau }}-1\right)\sigma_{bh}^{2}+\sigma_{sh}^{2}p_{s}^{\left(3\right)}}\exp\left(-\frac{2^{\frac{TR_{s}}{T-\tau }}-1}{\sigma_{sh}^{2}p_{s}^{\left(3\right)}}\right). \end{array}\right. \end{array}$$

In State0, the spectrum is idle and correctly detected with probability as $P_{1}=P\left(H_{0}\right)\left(1-p_{f}\right)$, and the SN transmits with power $p_{s}^{\left(0\right)}$. In State1, the spectrum is occupied and incorrectly detected as idle with probability as $P_{1}=P\left(H_{1}\right)\left(1-p_{d}\right)$, and the SN transmits with power $p_{s}^{\left(1\right)}$. In State2, the spectrum is idle and incorrectly detected as busy with probability $P_{2}=P\left(H_{0}\right)p_{f}$, and the SN transmits with power $p_{s}^{\left(2\right)}$. In State3, the spectrum is occupied and correctly detected with probability $P_{3}=P\left(H_{1}\right)p_{d}$, and the SN transmits with power $p_{s}^{\left(3\right)}$. Therefore, the average outage probability of the sensor network is:

(47) $$\begin{array}{cc} P_{out}^{s}= & P_{out}^{\left(0\right)}P_{0}+P_{out}^{\left(1\right)}P_{1}+P_{out}^{\left(2\right)}P_{2}+P_{out}^{\left(3\right)}P_{3}\\ = & \left(1-\exp\left(-\frac{2^{\frac{TR_{s}}{T-\tau }}-1}{\sigma_{sh}^{2}p_{s}^{\left(0\right)}}\right)\right)P\left(H_{0}\right)\left(1-p_{f}\right)\\ & +\left(1-\frac{\sigma_{bh}^{2}\sigma_{sh}^{2}p_{s}^{\left(1\right)}}{\left(2^{\frac{TR_{s}}{T-\tau }}-1\right)\sigma_{bh}^{2}+\sigma_{sh}^{2}p_{s}^{\left(1\right)}}\exp\left(-\frac{2^{\frac{TR_{s}}{T-\tau }}-1}{\sigma_{sh}^{2}p_{s}^{\left(1\right)}}\right)\right)P\left(H_{0}\right)p_{f}\\ & +\left(1-\exp\left(-\frac{2^{\frac{TR_{s}}{T-\tau }}-1}{\sigma_{sh}^{2}p_{s}^{\left(2\right)}}\right)\right)P\left(H_{1}\right)\left(1-p_{d}\right)\\ & +\left(1-\frac{\sigma_{bh}^{2}\sigma_{sh}^{2}p_{s}^{\left(3\right)}}{\left(2^{\frac{TR_{s}}{T-\tau }}-1\right)\sigma_{bh}^{2}+\sigma_{sh}^{2}p_{s}^{\left(3\right)}}\exp\left(-\frac{2^{\frac{TR_{s}}{T-\tau }}-1}{\sigma_{sh}^{2}p_{s}^{\left(3\right)}}\right)\right)P\left(H_{1}\right)p_{d}. \end{array}$$

## 5. Simulation Results

In this section, we will evaluate the performance of the primary network and CRSN. In the simulation, the frame duration *T* is set to be 50 ms. The path loss of the channel is set to be three. The SN’s average power and peak power are set to be 10 dB and 15 dB, respectively. The peak power of the PT is set to be 15 dB. In the simulation, we choose the simulation parameters according to the previous works on wireless sensor networks [47], cognitive radio networks [48] and CRSN [49,50,51]. In addition, these values are application-specific parameters and will vary according to the demands of the applications and constraints. The simulation assumptions, as well as other previous simulations will contribute to the development of the realistic CRSN architecture. In addition, changing some assumptions, such as the spectrum sensing method, will slightly affect the conclusions in this paper, and therefore, the obtained results in this paper reveal the general effects of the parameters on the performance in CRSN. For the proposed scheme, the critical parameters, such as channel idle probability, the spectrum sensing time, the power budget of the PT and the secrecy outage threshold, which will affect the performance of the proposed strategy, will be investigated in this section.

**Figure 2:**
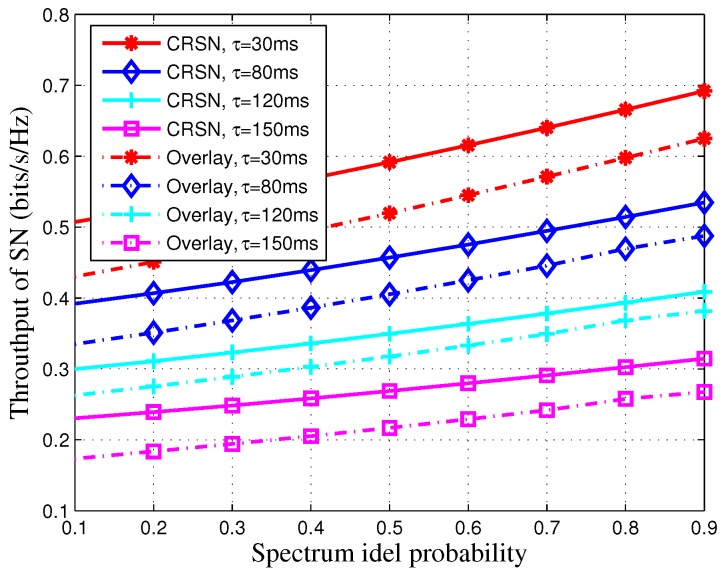
SN’s average throughput of the proposed strategy as a function of the spectrum idle probability. The transmit power of the primary transmit (PT) is set to $P_{b}=15$ dB, and the target secrecy outage probability is set to $P_{th}=0.1$.

In Figure 2, we plot the SN’s average throughput versus the spectrum idle probability $P\left(H_{0}\right)$. In this figure, we can observe that the SN’s average throughput is a monotonically increasing function with respect to $P\left(H_{0}\right)$. The reason is that the large spectrum idle probability indicates that there will be more interference-free spectrum access opportunities for the SN’s transmission, and the throughput of the CRSN will increase. In addition, more time allocated for spectrum sensing will result in less time for the SN’s transmission. Therefore, the average throughput of the SN will decrease. The throughput of the CRSN will decrease when a long time is allocated for spectrum sensing even through the detection probability will increase. Moreover, we also plot the traditional overlay scheme for contrast. Since the overlay scheme does not consider the cooperation between the primary network and the CRSN, the CRSN may be interfered by the PT when the occupied spectrum is detected as idle or miss the idle spectrum opportunity when the idle spectrum is detected as busy. Therefore, the throughput of the overlay scheme is lower than our proposed strategy.

**Figure 3:**
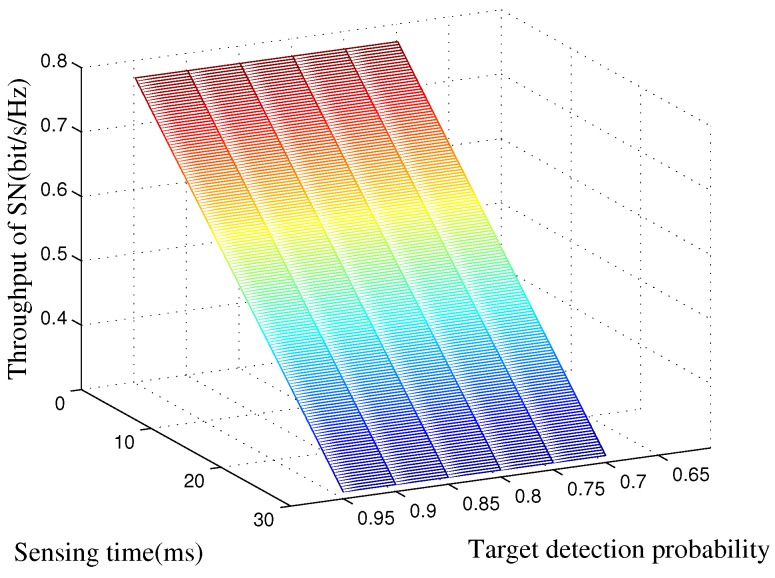
SN’s average throughput of the proposed strategy as a function of the spectrum sensing time and target detection probability. The spectrum idle probability is set to $P\left(H_{0}\right)$, the transmit power of the PT is set to $P_{b}=15$ dB, and the target secure outage probability is set to $P_{th}=0.1$.

In Figure 3, we show the SN’s average throughput of the proposed scheme versus the spectrum sensing time and the target detection probability. In this figure, we can observe that SN’s rate will decrease when more time is consumed for spectrum sensing. The reason is that when more time is allocated for spectrum sensing, there will be less time for the SN’s data transmission. Since $p_{s}^{\left(0\right)}=p_{s}^{\left(2\right)}$ and $p_{s}^{\left(1\right)}=p_{s}^{\left(3\right)}$, the average rate is $R_{s}=2P\left(H_{0}\right)E\left(R_{s}^{\left(0\right)}\right)+2P\left(H_{1}\right)E\left(R_{s}^{\left(1\right)}\right)$, which is not a function of the target detection probability. Therefore, the SN’s average rate will keep the same when the target detection probability changes.

**Figure 4:**
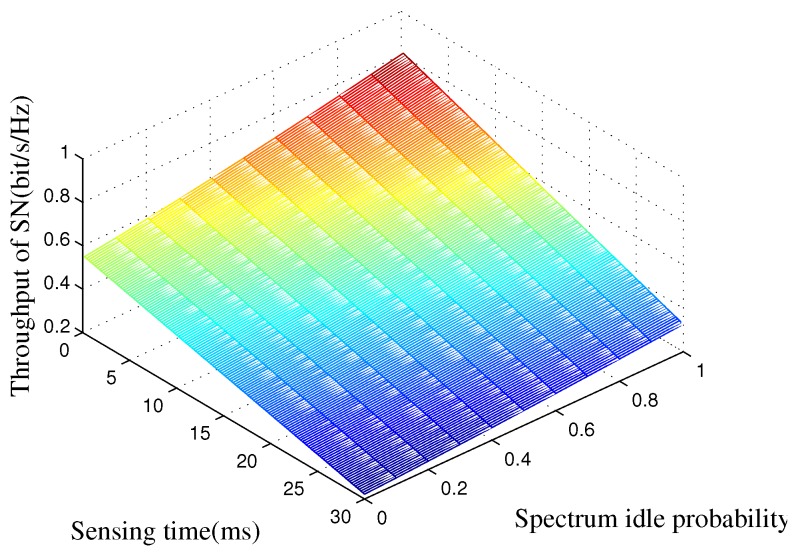
SN’s average throughput of the proposed strategy as a function of the spectrum sensing time and spectrum idle probability. The transmit power of the BS is set to $P_{p}=15$ dB, and the target secure outage probability is set to $P_{th}=0.1$.

In Figure 4, we show the SN’s average rate versus the spectrum sensing time *τ* and the spectrum idle probability $P\left(H_{0}\right)$. In this figure, we can observe that the SN’s average rate is a monotonically-increasing function with respect to $P\left(H_{0}\right)$. The reason is that a large value of the spectrum idle probability indicates that there will be more interference-free spectrum access opportunities for the SN’s transmission regardless of the security requirement of the primary network. In addition, the long spectrum sensing time indicates that the SN occupies a short time for information transmission, and the throughput will decrease.

**Figure 5:**
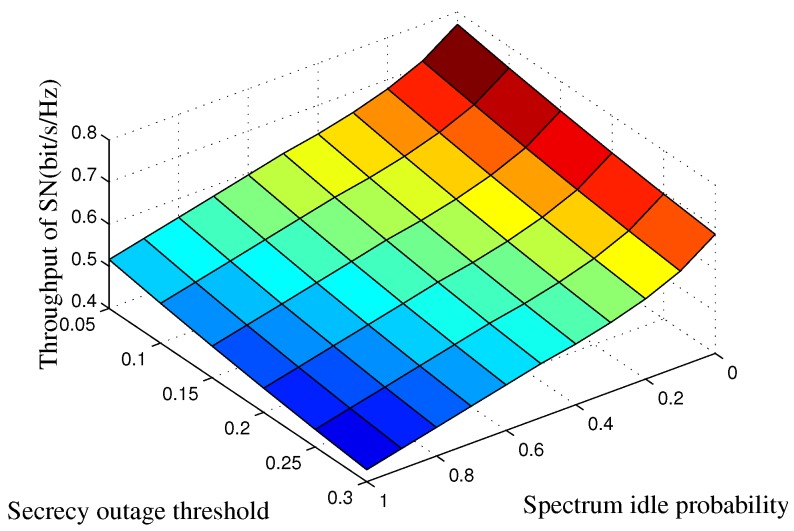
SN’s average throughput of the proposed strategy as a function of the target secrecy outage probability and spectrum idle probability. The transmit power of the PT is set to $P_{b}=15$ dB, and the spectrum sensing time is set to τ=1 ms.

In Figure 5, we show the SN’s average throughput versus the spectrum idle probability $P\left(H_{0}\right)$ and the target secrecy outage probability $P_{th}$. A small value of $P_{th}$ indicates that the secrecy outage probability constraint is stringent. Then, the SN has to spend more power to interfere with the eavesdropper and guarantee the secure transmission of the primary network. Therefore, the throughput of the SN will decrease. In addition, more power allocated for the SN’s data transmission will result in the increasing of the SN’s throughput. A large value of $P\left(H_{0}\right)$ indicates that there are more interference-free spectrum access opportunities regardless of the security requirement of the primary network. Under this condition, the SN can transmit with large power and achieve high transmission throughput.

**Figure 6:**
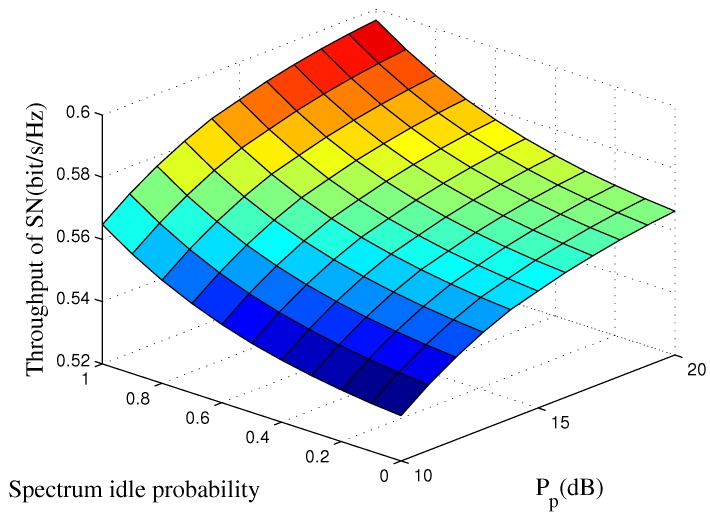
SN’s average throughput of the proposed strategy as a function of the PT’s transmit power $P_{b}$ and spectrum sensing time *τ*. The idle probability is set to $P\left(H_{0}\right)=0.8$, and the target secrecy outage probability is set to $P_{th}=0.1$.

In Figure 6, we show the SN’s average rate versus the PT’s transmit power $P_{b}$ and the spectrum idle probability $P\left(H_{0}\right)$. A large value $P_{b}$ indicates that there will be more power consumed for the primary network to guarantee its secure transmission. Then, the SN will consume less power to cooperate with the primary network, which contributes to increasing the SN’s throughput. In addition, when $P_{b}$ is large, the detection probability will improve, and the CRSN can fully utilize the spectrum and optimally control its transmit power to maximize its throughput.

## 6. Conclusions

In this paper, we proposed a cooperative secure transmission strategy, which would benefit both the primary network and the CRSN. In this strategy, the SN’s transmission would protect primary information security, and as a reward, limited spectrum sensing error probability was permitted. Based on the primary activities and spectrum sensing results, there were four system states in the system. In each state, we analyzed the SN’s transmission rate and the primary secrecy rate and, then, optimally allocated the SN’s transmit power to maximize the SN’s average rate under the constraint of the maximum permitted primary secrecy outage probability. In addition, we gave further discussion for the power allocation strategy and the performance tradeoff between the transmission rate of the CRSN, and the secrecy outage probability of the primary network was investigated. Moreover, we investigated the primary secrecy rate for the asymptotic scenarios and derived closed-form expression of the SN’s transmission outage probability. Simulation results showed that: (1) the performance of the SN’s average throughput in the proposed strategy outperformed the conventional overlay strategy; (2) the primary confidential messages were protected, and the QoS requirement of the CRSN is satisfied.

## Figures and Tables

**Figure A1:**
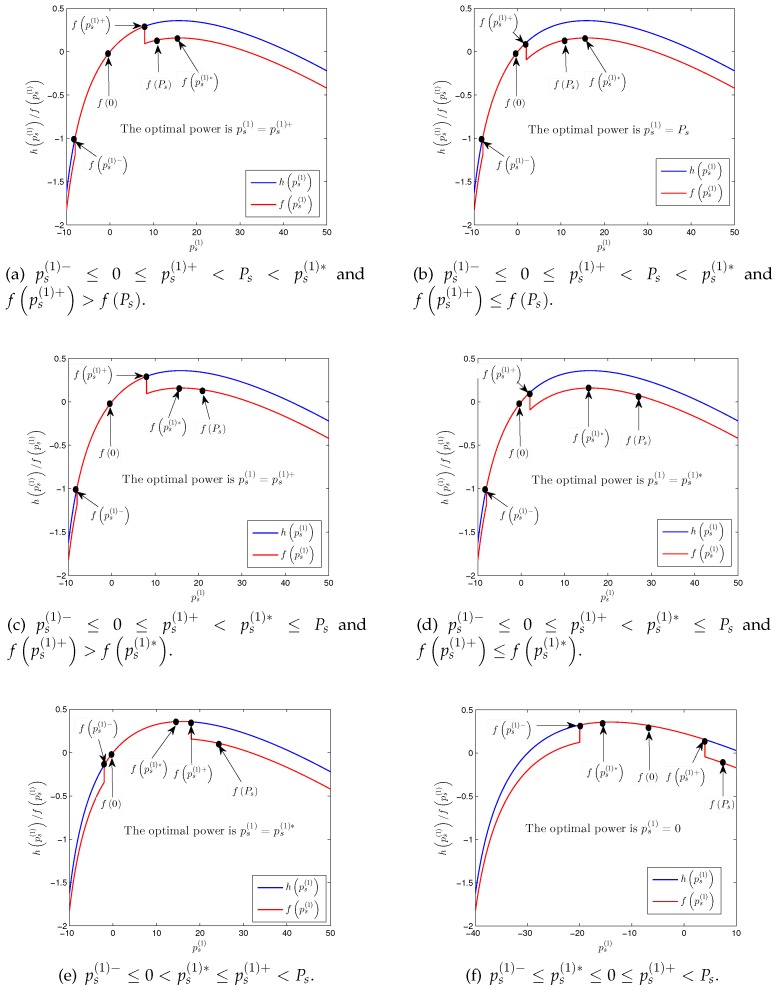
Illustration of $p_{s}^{\left(1\right)-}\leq 0\leq p_{s}^{\left(1\right)+}<P_{s}$.

## Appendix A. The Derivation Process of $p_{s}^{\left(1\right)}$

Set $h\left(p_{s}^{\left(1\right)}\right)=R_{s}^{\left(1\right)}P_{1}+\lambda \left(P_{av}-\frac{T-\tau }{T}P_{1}p_{s}^{\left(1\right)}\right)+\mu P_{th}$ and $f\left(p_{s}^{\left(1\right)}\right)=h\left(p_{s}^{\left(1\right)}\right)-\mu \varphi \left(p_{s}^{\left(1\right)}\right)$. Then, there are six cases in the derivation of the optimal transmit power. In Figure A1, we only show the first case, and the other five cases are similar to this case.

- $p_{s}^{\left(1\right)-}\leq 0\leq p_{s}^{\left(1\right)+}<P_{s}$: If $p_{s}^{\left(1\right)-}\leq 0\leq p_{s}^{\left(1\right)+}<P_{s}<p_{s}^{\left(1\right)*}$ and $f\left(p_{s}^{\left(1\right)+}\right)>f\left(P_{s}\right)$, the optimal transmit power is $p_{s}^{\left(1\right)}=p_{s}^{\left(1\right)+}$, which is shown in Figure A1a. Otherwise, if $f\left(p_{s}^{\left(1\right)+}\right)\leq f\left(P_{s}\right)$, the optimal transmit power is $p_{s}^{\left(1\right)}=P_{s}$, which is shown in Figure A1b. If $p_{s}^{\left(1\right)-}\leq 0\leq p_{s}^{\left(1\right)+}<p_{s}^{\left(1\right)*}\leq P_{s}$ and $f\left(p_{s}^{\left(1\right)+}\right)>f\left(p_{s}^{\left(1\right)*}\right)$, the optimal transmit power is $p_{s}^{\left(1\right)}=p_{s}^{\left(1\right)+}$, which is shown in Figure A1c. Otherwise, if $f\left(p_{s}^{\left(1\right)+}\right)\leq f\left(p_{s}^{\left(1\right)*}\right)$, the optimal transmit power is $p_{s}^{\left(1\right)}=p_{s}^{\left(1\right)*}$, which is shown in Figure A1d. If $p_{s}^{\left(1\right)-}\leq 0<p_{s}^{\left(1\right)*}\leq p_{s}^{\left(1\right)+}<P_{s}$, the optimal transmit power is $p_{s}^{\left(1\right)}=p_{s}^{\left(1\right)*}$, which is shown in Figure A1e. If $p_{s}^{\left(1\right)-}\leq p_{s}^{\left(1\right)*}\leq 0\leq p_{s}^{\left(1\right)+}<P_{s}$, the optimal transmit power is $p_{s}^{\left(1\right)}=0$, which is shown in Figure A1f.
- $p_{s}^{\left(1\right)-}\leq 0\leq P_{s}\leq p_{s}^{\left(1\right)+}$: If $p_{s}^{\left(1\right)-}\leq 0\leq P_{s}\leq p_{s}^{\left(1\right)+}<p_{s}^{\left(1\right)*}$ or $p_{s}^{\left(1\right)-}\leq 0\leq P_{s}<p_{s}^{\left(1\right)*}\leq p_{s}^{\left(1\right)+}$, the optimal transmit power is $p_{s}^{\left(1\right)}=P_{s}$. If $p_{s}^{\left(1\right)-}\leq 0<p_{s}^{\left(1\right)*}\leq P_{b}\leq p_{s}^{\left(1\right)+}$, the optimal transmit power is $p_{s}^{\left(1\right)}=p_{s}^{\left(1\right)*}$. If $p_{s}^{\left(1\right)-}<p_{s}^{\left(1\right)*}\leq 0\leq P_{s}\leq p_{s}^{\left(1\right)+}$, the optimal transmit power is $p_{s}^{\left(1\right)}=0$.
- $0\leq p_{s}^{\left(1\right)-}\leq P_{s}<p_{s}^{\left(1\right)+}$ : If $0\leq p_{s}^{\left(1\right)-}\leq P_{s}<p_{s}^{\left(1\right)+}<p_{s}^{\left(1\right)*}$ or $0\leq p_{s}^{\left(1\right)-}\leq P_{s}<p_{s}^{\left(1\right)*}\leq p_{s}^{\left(1\right)+}$, the optimal transmit power is $p_{s}^{\left(1\right)}=P_{s}$. If $0\leq p_{s}^{\left(1\right)-}<p_{s}^{\left(1\right)*}\leq P_{s}<p_{s}^{\left(1\right)+}$, the optimal transmit power is $p_{s}^{\left(1\right)}=\left(1\right)*$. If $0<p_{s}^{\left(1\right)*}\leq p_{s}^{\left(1\right)-}\leq P_{s}<p_{s}^{\left(1\right)+}$ and $f\left(p_{s}^{\left(1\right)*}\right)>f\left(p_{s}^{\left(1\right)-}\right)$, the optimal transmit power is $p_{s}^{\left(1\right)}=p_{s}^{\left(1\right)*}$. Otherwise, if $f\left(p_{s}^{\left(1\right)*}\right)\leq f\left(p_{s}^{\left(1\right)-}\right)$, the optimal transmit power is $p_{s}^{\left(1\right)}=p_{s}^{\left(1\right)-}$.
- $0\leq p_{s}^{\left(1\right)-}<p_{s}^{\left(1\right)+}\leq P_{b}$: If $0\leq p_{s}^{\left(1\right)-}<p_{s}^{\left(1\right)+}\leq P_{s}<p_{s}^{\left(1\right)*}$ and $f\left(p_{s}^{\left(1\right)+}\right)>f\left(P_{s}\right)$, the optimal transmit power is $p_{s}^{\left(1\right)}=p_{s}^{\left(1\right)+}$. Otherwise, if $f\left(p_{s}^{\left(1\right)+}\right)\leq f\left(P_{s}\right)$, the optimal transmit power is $p_{s}^{\left(1\right)}=P_{s}$. If $0\leq p_{s}^{\left(1\right)-}<p_{s}^{\left(1\right)+}<p_{s}^{\left(1\right)*}\leq P_{s}$ and $f\left(p_{s}^{\left(1\right)+}\right)>f\left(\left(1\right)*\right)$, the optimal transmit power is $p_{s}^{\left(1\right)}=p_{s}^{\left(1\right)+}$. Otherwise, if $f\left(p_{s}^{\left(1\right)+}\right)\leq f\left(p_{s}^{\left(1\right)*}\right)$, the optimal transmit power is $p_{s}^{\left(1\right)}=p_{s}^{\left(1\right)*}$. If $0\leq p_{s}^{\left(1\right)-}<p_{s}^{\left(1\right)*}\leq p_{s}^{\left(1\right)+}\leq P_{s}$, the optimal transmit power is $p_{s}^{\left(1\right)}=p_{s}^{\left(1\right)*}$. If $0<p_{s}^{\left(1\right)*}\leq p_{s}^{\left(1\right)-}<p_{s}^{\left(1\right)+}\leq P_{s}$ and $f\left(p_{s}^{\left(1\right)-}\right)>f\left(p_{s}^{\left(1\right)*}\right)$, the optimal transmit power is $p_{s}^{\left(1\right)}=p_{s}^{\left(1\right)*}$. Otherwise, if $f\left(p_{s}^{\left(1\right)*}\right)\leq f\left(p_{s}^{\left(1\right)-}\right)$, the optimal transmit power is $p_{s}^{\left(1\right)}=p_{s}^{\left(1\right)-}$.
- $p_{s}^{\left(1\right)-}<p_{s}^{\left(1\right)+}\leq 0<P_{s}$: If $p_{s}^{\left(1\right)-}<p_{s}^{\left(1\right)+}\leq 0<P_{s}<p_{s}^{\left(1\right)*}$, the optimal transmit power is $p_{s}^{\left(1\right)}=P_{s}$. If $p_{s}^{\left(1\right)-}<p_{s}^{\left(1\right)+}\leq 0<p_{s}^{\left(1\right)*}\leq P_{s}$, the optimal transmit power is $p_{s}^{\left(1\right)}=p_{s}^{\left(1\right)*}$. If $p_{s}^{\left(1\right)-}<p_{s}^{\left(1\right)+}\leq p_{s}^{\left(1\right)*}\leq 0<P_{s}$ or $p_{s}^{\left(1\right)-}\leq p_{s}^{\left(1\right)*}<p_{s}^{\left(1\right)+}\leq 0<P_{s}$, the optimal transmit power is $p_{s}^{\left(1\right)}=0$.
- $0<P_{b}\leq p_{s}^{\left(1\right)-}<p_{s}^{\left(1\right)+}$: If $0<P_{s}\leq p_{s}^{\left(1\right)-}<p_{s}^{\left(1\right)+}<p_{s}^{\left(1\right)*}$, $0<P_{s}\leq p_{s}^{\left(1\right)-}<p_{s}^{\left(1\right)*}\leq p_{s}^{\left(1\right)+}$ or $0<P_{s}<p_{s}^{\left(1\right)*}\leq p_{s}^{\left(1\right)-}<p_{s}^{\left(1\right)+}$, the optimal transmit power is $p_{s}^{\left(1\right)}=P_{s}$. If $0\leq p_{s}^{\left(1\right)*}\leq P_{b}\leq p_{s}^{\left(1\right)-}<p_{s}^{\left(1\right)+}$, the optimal transmit power is $p_{s}^{\left(1\right)}=p_{s}^{\left(1\right)*}$.

Therefore, the $p_{s}^{\left(1\right)}$ for this scenario is derived.
